# Checkpoint immunotherapy is associated with preferential activation of tumor antigen–specific CD4^+^ T cells in MDS

**DOI:** 10.1016/j.bneo.2025.100106

**Published:** 2025-04-25

**Authors:** Elizabeth A. Griffiths, Pragya Srivastava, Eduardo Cortes Gomez, Junko Matsuzaki, Kunle Odunsi, Laura W. Dillon, Devdeep Mukherjee, Christopher S. Hourigan, Jacqueline Peng, Shovik Bandyopadhyay, Kai Tan, Kristopher M. Attwood, Joseph B. Kuechle, Prashant K. Singh, Jianmin Wang, Michael J. Nemeth

**Affiliations:** 1 Department of Medicine, Roswell Park Comprehensive Cancer Center, Buffalo, NY; 2 Department of Biostatistics and Bioinformatics, Roswell Park Comprehensive Cancer Center, Buffalo, NY; 3 Department of Immunology, Center for Immunotherapy, Roswell Park Comprehensive Cancer Center, Buffalo, NY; 4 Department of Obstetrics and Gynecology, University of Chicago Medicine Comprehensive Cancer Center, Chicago, IL; 5 Laboratory of Myeloid Malignancies, Hematology Branch, National Heart, Lung, and Blood Institute, Bethesda, MD; 6 Fralin Biomedical Research Institute, Virginia Tech Fralin Biomedical Research Institute Cancer Research Center, Washington, DC; 7 Graduate Group in Genomics and Computational Biology, Perelman School of Medicine, University of Pennsylvania, Philadelphia, PA; 8 Cellular and Molecular Biology Graduate Group, Perelman School of Medicine, University of Pennsylvania, Philadelphia, PA; 9 Department of Pediatrics, Children's Hospital of Philadelphia, University of Pennsylvania Perelman School of Medicine, Philadelphia, PA; 10 Department of Orthopedics, University at Buffalo, Buffalo, NY; 11 Department of Cancer Genetics and Genomics, Roswell Park Comprehensive Cancer Center, Buffalo, NY; 12 Department of Immunology, Roswell Park Comprehensive Cancer Center, Buffalo, NY

## Abstract

A growing body of literature suggests that the efficacy of DNA hypomethylating agents are mediated via activation of antitumor immune mechanisms. Based upon this hypothesis, early phase trials combining immune checkpoint inhibitors (ICIs) with azacitidine in patients with myelodysplastic syndrome (MDS)/acute myeloid leukemia (AML) were undertaken, but clinical and immunologic efficacy have proven disappointing. In these studies, the lack of antigen specificity made systematic assessment of the anti-MDS immune response challenging. We hypothesized that combining vaccination against the New York esophageal squamous cell carcinoma 1 (NY-ESO-1) tumor antigen with decitabine and an ICI would allow us to understand antigen-specific immune responses in patients with MDS. To test this hypothesis, we developed an investigator-initiated phase 1 trial in transplant-ineligible patients with MDS/low blast count AML incorporating the anti–programmed cell death protein-1 (PD-1) ICI nivolumab. All patients developed NY-ESO-1–specific CD4^+^ T-cell responses associated with upregulation of anti–PD-1 immunotherapy gene signatures in the CD4^+^ T-cell population. Patients had reduced numbers of conventional dendritic cells marked by high expression of CD141 (cDC1), a population critical for successful responses to immunotherapy. cDC1 from patients with MDS showed reduced expression of genes that are key for optimal T-cell activation and expansion. These results suggest that immunotherapy efficacy may vary according to the function of the myeloid immunologic milieu in patients with MDS. Approaches to augment the number and function of cDC1 populations in myeloid disease might overcome this defect and enhance the efficacy of immunotherapy for patients with MDS. This trial was registered at www.ClinicalTrials.gov as #NCT03358719.

## Key Points

• Antigen-specific vaccination in the context of checkpoint immunotherapy reveals preferential activation of CD4^+^ T cells in patients with MDS.
• Appreciation of the altered immune environment in MDS is important to optimize immunotherapy in this disease.

## Visual Abstract

**Figure:**
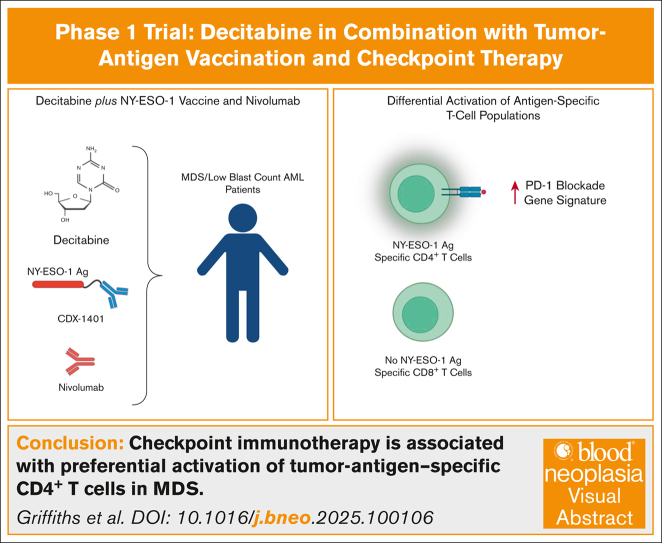

## Introduction

Myelodysplastic syndromes (MDS) are diseases of bone marrow failure in which insufficient production of mature blood cells results in life-threatening complications from anemia, thrombocytopenia, and increased risk of infections. For a substantial portion of those with higher-risk disease, transformation to acute myeloid leukemia (AML) is inevitable and associated with dismal survival.^1^ Patients with intermediate and higher-risk MDS are generally offered treatment with the DNA hypomethylating agents (HMA) azacitidine and decitabine. HMAs can improve quality-of-life and mitigate transfusion dependence in association with a reduced rate of AML transformation.^2^^,^^3^ However, <50% of patients treated show clinical response to therapy and loss of response is inevitable.^4^^,^^5^ Survival in nonresponders and those who progress after initial response is exceedingly poor, with long-term survival observed only in those who successfully undergo allogeneic stem cell transplant.^6^ There is an urgent need to develop new approaches that complement standard-of-care HMA therapy for this underserved patient population.

Recent studies have revealed that the clinical efficacy of HMAs may be mediated through activation of antitumor immune mechanisms. HMAs induce expression of tumor-associated antigens and factors involved in antigen processing and presentation in tumor cells.^7^, ^8^, ^9^, ^10^, ^11^, ^12^, ^13^ HMAs increase expression of endogenous retroviral sequences, which could activate an immune response against viral RNAs and antigens.^14^, ^15^, ^16^ HMAs also induce expression of proinflammatory cytokines and chemokines.^17^ Thus, the combination of immunotherapies with standard-of-care HMA therapy is an active area of clinical research in patients with MDS. One field of interest is the use of immune checkpoint inhibitors (ICIs) such as nivolumab, a human monoclonal antibody against programmed cell death protein-1 (PD-1).^18^ PD-1 is expressed on the surface of T cells from patients with MDS/AML.^19^^,^^20^ We and others have observed that the ligand for PD-1, programmed death ligand-1, is expressed on blasts from patients with MDS/AML.^13^^,^^19^ However, recent early phase trials combining ICIs with azacitidine in patients with MDS/AML have demonstrated limited efficacy in terms of both clinical and immunologic response.^21^, ^22^, ^23^, ^24^ Correlative studies have been limited to descriptive immunologic gene signatures and changes in the number of immune cell populations and have suggested successful activation of immune populations in responding patients.^24^^,^^25^ Because none of these studies examined the response to therapy in an antigen-specific manner, understanding of the anti-MDS immune milieu has been limited. This is particularly important in MDS as the disease involves dysplasia of multiple immune cell populations, including populations of antigen presenting cells that regulate antitumor T-cell responses.^26^^,^^27^

One approach to understanding the ability of the immune milieu in MDS to respond to ICI is to examine responses in a tumor antigen–specific manner. New York esophageal squamous cell carcinoma 1 (NY-ESO-1) is a well-characterized immunogenic tumor antigen whose expression is normally suppressed in MDS and other myeloid cancers through dense promoter hypermethylation.^7^^,^^11^^,^^13^^,^^28^^,^^29^ We observed induced expression of NY-ESO-1 in primary blasts from patients receiving standard-of-care HMA therapy. The level of NY-ESO-1 expression was sufficient to activate cytotoxic responses in NY-ESO-1–specific CD8^+^ T cells.^13^ Subsequently, we treated patients with MDS in a phase 1 study with a combination of vaccination against NY-ESO-1 with standard decitabine.^30^ This combination was safe, and most patients developed NY-ESO-1–specific T-cell responses following vaccination.

We hypothesized that combining anti-NY-ESO-1 vaccination with decitabine and nivolumab would enhance the immune response to vaccination and allow us to better understand tumor antigen-specific immunity in patients with MDS. To test our hypothesis, we developed a second investigator-initiated phase 1 trial in transplant-ineligible patients with MDS/low blast count AML which integrated nivolumab into our established combination of decitabine plus NY-ESO-1 vaccination. We found in a limited cohort that patients developed NY-ESO-1–specific CD4^+^ T-cell responses, and these responses were associated with upregulation of an anti–PD-1 immunotherapy gene signature in the CD4^+^ T-cell compartment. Compared with healthy individuals, patients had reduced numbers of CD141^Hi^ conventional dendritic cells (cDC1s), a population critical for successful responses to immunotherapy.^27^ cDC1s from patients with MDS also showed reduced expression of genes that are key mediators of optimal T-cell responses. These results suggest that the use of immunotherapy to induce effective, cytotoxic antitumor T-cell responses depends upon the myeloid immunologic milieu in patients with MDS as described in multiple studies in the context of solid tumors.^31^ This critical finding suggests that approaches to augment the number and function of specific dendritic cell populations could enhance the efficacy of immunotherapy.

## Methods

### Study design

This was an open-label, nonrandomized single-center phase 1 study of NY-ESO-1 vaccine (CDX-1401 [1 mg; Celldex Therapeutics] + polyinosinic-polycytidylic acid-poly-l-lysine carboxymethylcellulose [1.8 mg, Hiltonol; Oncovir]) administered in combination with standard dose decitabine (20 mg/m^2^ per day × 5 days) and nivolumab (3 mg/kg every 2 weeks) in patients with MDS or low blast count AML. Planned study treatments included 5 vaccinations and decitabine/nivolumab cycles to be given on schedule every 4 weeks. The study ended after cycle 4, on day 29. The primary objective of this study was safety. The secondary objective was to assess immune and molecular epigenetic responses following combination therapy. Exploratory objectives included the overall response rate (complete response, partial response with hematologic improvement), overall survival, and progression-free survival as descriptive characteristics. Eight patients were enrolled and treated in the study (ClinicalTrials.gov identifier: NCT 03358719) which was conducted in accordance with the Declaration of Helsinki and approved by the Roswell Park Comprehensive Cancer Center (Roswell Park) Internal Review Board (IRB). All patients provided written informed consent.

This study was a modified 3+3 design. All patients were accrued and treated at dose level 1 (DL1). Decitabine was administered at 20 mg/m^2^ per day, IV, over 1 hour on days 1 to 5, followed by 23 days without decitabine. The dose-limiting toxicity (DLT) window began on day −14 of cycle 1 and ended on day 1 of cycle 2. Related grade ≥3 nonhematologic toxicities would have been considered dose limiting. If none or 1 of the first 3 patients had a DLT, then 3 more patients were to be enrolled at this dose. Because ≤1 of the first 6 patients had a DLT, DL1 was declared the maximum administered dose. The study design provided for dose de-escalation (to DL-1) should there be unexpected toxicities at the standard doses for all administered drugs, but this was not necessary.

### Patient samples

For patients enrolled in the study, peripheral blood was obtained at baseline, twice weekly, and at the end of the study (EOS). CD11b^+^ myeloid cells were isolated from peripheral mononuclear cells (PBMCs) by Ficoll centrifugation followed by CD11b microbeads as per manufacturer instructions (Miltenyi Biotec). Bone marrow was collected before the initiation of study therapy (“baseline”) and after the fourth cycle of decitabine. Patients enrolled in this study are designated DVN to indicate the triple combination of decitabine, vaccine, and nivolumab. Additional retrospectively analyzed specimens from individuals with MDS treated with standard-of-care decitabine, on our prior clinical trial,^32^ or healthy donors (HDs; defined as absence of hematologic malignancy) were included for comparative analyses. Deidentified peripheral blood and bone marrow samples from these patients and HDs were collected in accordance with the Declaration of Helsinki and their use approved under IRB-approved protocols at Roswell Park and University at Buffalo. Samples from our previous study were designated DV (decitabine and vaccine) and samples from patients receiving single-agent decitabine are designated D (decitabine). Bone marrow cells from deidentified HDs were obtained from remnant tissue following hip-replacement surgery. All patients had provided written informed consent for collection of sample material and its retrospective use under IRB-approved protocols at Roswell Park.

### Analysis of NY-ESO-1–specific immune responses

To measure the frequency of NY-ESO-1–specific T cells, enzyme-linked immunospot (ELISPOT) assays were performed on CD4^+^ and CD8^+^ T lymphocytes isolated from PBMCs collected at baseline and at the EOS.^10^^,^^30^ To measure anti–NY-ESO-1 antibody titers, enzyme-linked immunosorbent assay was performed using patient sera collected at baseline and at EOS.^28^^,^^32^ Further details are available in the supplemental Methods.

### Single-cell RNA sequencing (scRNA-seq) and analysis

Analyses of data that passed quality control were performed using Seurat (version 4.1.0) single-cell analysis R package.^33^ SingleR (version 1.6.1) was used to annotate individual immune cell types using the Blueprint-Encode database.^34^ For downstream visualization, data were dimension-reduced via Uniform Manifold Approximation and Projection (UMAP) and clustered (Louvain algorithm). Gene set enrichment analysis (GSEA) is performed on comparisons of interest using GSEA preranked procedure from GSEA (version 3.0 beta).^35^ Negative log*P* values are multiplied by the sign of the log2FC and entered as ranked list input. Hallmark and C2CP gene symbol sets from MSigDB (version 7.1) were employed for these analyses. Enriched pathways are defined as an adjusted *P* value ≤.01. Further details are available in the supplemental Methods.

## Results

### Clinical and molecular responses to DVN immunotherapy

**Figure 1.**
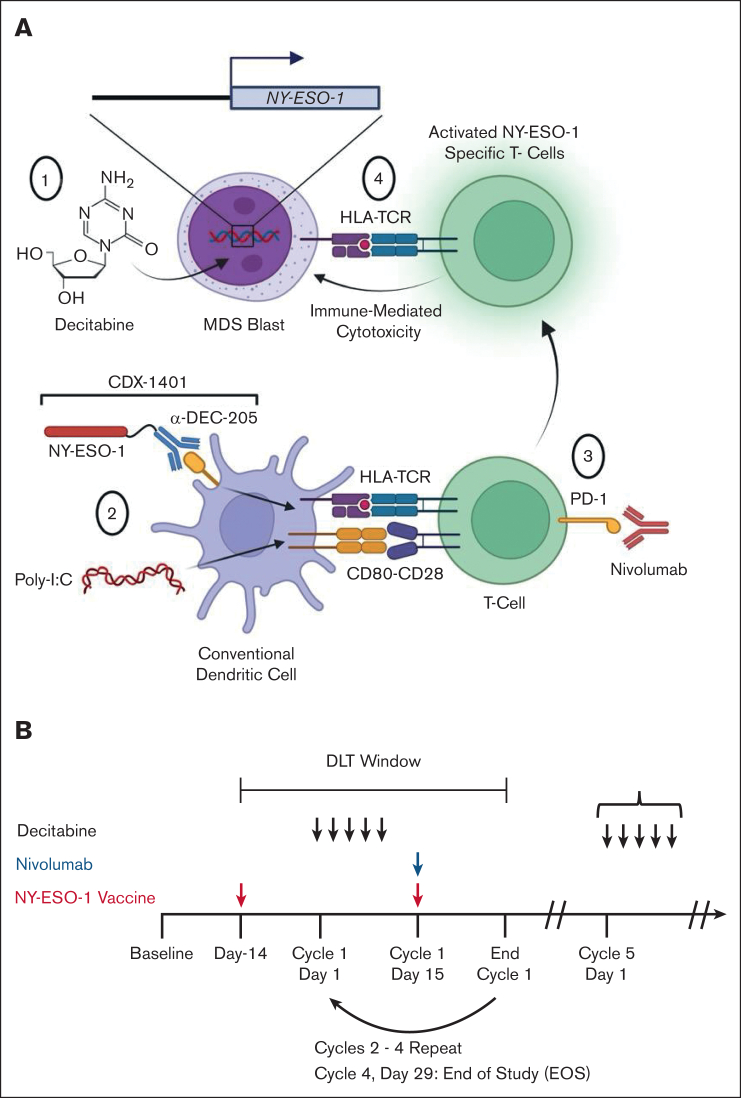
**Schematic of clinical trial design.** (A) Proposed model of the effects of decitabine, NY-ESO-1 vaccination (CDX-1401), and nivolumab in patients in the study. (1) Decitabine induces expression of NY-ESO-1 in patient blasts. (2) cDCs take up CDX-1401 through the DEC-205 receptor, process it, and present the full-length NY-ESO-1 protein as an antigen. Poly-ICLC activates DCs and induces expression of costimulatory molecules such as CD80. DCs activate T cells through interactions between antigen-bound HLA and the TCR and costimulatory molecules (such as CD80-CD28). (3) Administration of nivolumab leads to reinvigoration of NY-ESO-1–specific T cells. (4) These reinvigorated T cells cause cytotoxicity of NY-ESO-1–expressing blasts. Both CD8^+^ and CD4^+^ T cells are hypothesized to play a role in the immunologic response to therapy; for simplicity, the figure shown here focuses on the CD8^+^ T-cell response. (B) Schematic diagram showing the treatment schedule of decitabine, vaccine, and nivolumab. Initial responses to treatment were assessed during the DLT window. Throughout the study, serial peripheral blood, bone marrow, and plasma samples were collected. Poly(I:C), polyinosinic-polycytidylic acid; TCR, T-cell receptor.

To test the hypothesis that an ICI could enhance the development of tumor antigen–specific immune responses, we performed a phase 1 study in which patients received the anti–PD-1 antibody nivolumab (3 mg/kg) in addition to our previously established combination of decitabine and NY-ESO-1 vaccination (ClinicalTrials.gov identifier: NCT 03358719; Figure 1A). We selected decitabine for this study based on our prior work demonstrating that this agent was more potent at inducing NY-ESO-1 expression compared to azacitidine.^11^ The vaccine (CDX-1401) is a full-length NY-ESO-1 tumor antigen conjugated to a fully human monoclonal antibody with specificity for the dendritic cell receptor DEC-205 combined with poly-ICLC (Hiltonol).^30^^,^^36^ The use of a full-length antigen means that the vaccine is HLA unrestricted. The anti–DEC-205 antibody enables uptake of the full-length NY-ESO-1 by DEC-205^+^ cells which are predominantly DCs. This vaccine construct was selected based on its superior ability to activate antigen-specific CD8^+^ and CD4^+^ T-cell responses compared to nontargeted antigens.^37^ Poly-ICLC is a clinical grade synthetic, nuclease-resistant, hydrophilic complex of polyinosinic-polycytidylic acid (poly[I:C]) that stimulates the immune system by mimicking viral double-stranded RNA and binding to toll-like receptor 3 (TLR3). The first vaccination was administered 2 weeks before the start of decitabine to initiate the generation of anti–NY-ESO-1 immune responses before upregulation of NY-ESO-1 expression (Figure 1B). Subsequent vaccinations were administered on day 15 of each cycle during cycles 1 to 4. Patients received nivolumab at a dose of 3 mg/kg on day 15 of each decitabine cycle.

The clinical characteristics of the patient cohort are described in Table 1, and additional details on cytogenetics and long-term follow-up are provided supplemental Table 1. Eight patients were enrolled in the study, 7 of whom received at least 1 cycle of DVN therapy. All were treated at DL1. Adverse events were as expected for treatment with single-agent decitabine. Study related adverse events are summarized in Table 2 and were manageable with standard approaches. Patient 2DVN received a single vaccination and exhibited multisystem organ failure due to pneumonia, which resulted in their coming off study before decitabine therapy. Patient 4DVN had intermediate-risk MDS with low blasts but a complex karyotype and profound transfusion-dependent pancytopenia at study enrollment. This patient came off study after cycle 1 due to grade 4 rash related to nivolumab. Upon recovery from the rash, a bone marrow biopsy for EOS purposes showed normal cytogenetics and the patient had trilineage count recovery (supplemental Table 1). Of the 7 patients who completed at least 1 study cycle, 2 patients showed a complete response, 4 patients showed stable disease, and 1 patient showed progressive disease. We observed modest changes in the variant allele frequency for the predominant mutations in PBMC samples collected at baseline vs EOS (supplemental Table 2). These data suggest that patients on study exhibited a modest clinical response to DVN therapy.

**Table 1.** **Overview of clinical characteristics of patients enrolled in a phase 1 trial of decitabine given in combination with nivolumab in conjunction with vaccination against NY-ESO-1 (****ClinicalTrials.gov****identifier: NCT03358719)**

Patient	Age, y	Pathological diagnosis	IPSS-R	Baseline cytogenetics	EOS cytogenetics	Baseline BM blasts	EOS BM blasts	Study cycles completed	Best response on study
1DVN	74	MDS-MLD	Very high (6.5)	Complex; >3 abnormalities	NA	3	NA	2	PD
2DVN	71	AML-MRC	NA	47, XY, +8 [20]	NA	25	NA	0	NA
3DVN	85	MDS-EB-2	Int (4)	45,X,-Y [5] 46,XY [21]	45,X,-Y [4] 46,XY [16]	27	5	4	CR
4DVN	56	t-MDS	Int (3)	Complex; >2 abnormalities	46,XX [19]	1	1	1	CR
5DVN	80	AML-MRC	High (5)	46,XY [5]	46,XY [20]	22	9	4	SD
6DVN	72	MDS-MLD	Very high (6.5)	Complex; >3 abnormalities, >2 clones	Complex; >3 abnormalities, >2 clones	2	2	4	SD
7DVN	69	MDS-SLD	Int (3.5)	46,XX [20]	NA	3	NA	2	SD
8DVN	66	MDS-EB-2	Very high (8)	Complex; >3 abnormalities, >2 clones	Complex; >2 abnormalities, >1 clones	10	5	4	SD

Patients are marked by an ID number. Patient 1DVN completed 2 cycles of study before electing to discontinue treatment and there was insufficient material for all correlative assessments. Patient 2DVN received a single NY-ESO-1 vaccination and was evaluated for vaccine toxicity but came off study before receiving any cytotoxic chemotherapy. Immunologic assessments were performed for patients 3-8DVN on study. IPSS-R scores (shown in parentheses) are based on the criteria described in Greenberg et al.^38^ Best responses were annotated using modified Cheson criteria for MDS.^39^ A complete description of cytogenetics and long-term clinical follow-up are shown in supplemental Table 2.

BM, bone marrow; CR, complete remission; MDS-EB-2, myelodysplastic syndrome with excess blasts 2; Int, intermediate; IPSS-R, Revised iInternational Prognostic Scoring System; MDS-MLD, myelodysplastic syndrome with multilineage dysplasia; AMLMRC, acute myeloid leukemia with myelodysplasia-related changes; NA, not available; MDS-SLD, myelodysplastic syndrome with single lineage dysplasia; SD, stable disease; t-MDS, therapy-related myelodysplastic syndrome.

**Table 2.** **Patient safety data**

N = 8 patients	Grades 1-2	Grade ≥3
**Possible** **immune related**		
Rash/immune system disorder	4	3
Colitis	0	1
Cough/pneumonitis	4	0
LFT elevation	5	0
**Cytopenias**		
Anemia	4	4
Thrombocytopenia	0	2
Neutropenia	0	1
**Other**		
Chills	2	0
Constipation/abdominal pain	4	0
Fatigue	5	1
Infection∗	4	9
Injection site reaction	3	0
GI bleeding	2	0
Mucositis	3	1
Myalgia	3	1

Grading for adverse events was recorded throughout the study and assigned by the investigators according to the Common Terminology Criteria for Adverse Events version 4.0.

GI, gastrointestinal; LFT, -liver function test.

∗ Some patients enrolled in study had multiple adverse events due to infection.

### DVN therapy induces development of NY-ESO-1–specific CD4^+^ T-cell responses

Compared to baseline levels, all patients showed hypomethylation of the *NY-ESO-1* promoter and the methylation nadir occurred between days 8 and 15 of each decitabine cycle (supplemental Figure 1). There was no expression of NY-ESO-1 at baseline (supplemental Figure 2); 3 of 7 patients exhibited an increased expression of NY-ESO-1 in myeloid peripheral blood cells following the start of decitabine treatment. It is possible that other patients expressed levels of NY-ESO-1 below the limit of detection for this assay. Analyses of NY-ESO-1–specific immune responses were determined for 6 patients on study (3-8DVN). We performed enzyme-linked immunosorbent assay and ELISPOT analyses to determine NY-ESO-1–specific humoral and cellular immune responses (Table 3). No patients exhibited NY-ESO-1–specific antibodies at baseline and only 1 patient developed NY-ESO-1–specific antibodies by EOS. For each patient, ELISPOT analyses were performed at EOS or, if the patient came off-study early, the latest available time point. This approach was used to capture the likeliest maximum possible vaccine response. There were undetectable numbers of NY-ESO-1–specific CD4^+^ and CD8^+^ T cells in all patients at baseline. By EOS, 4 of 5 (80%) patients exhibited NY-ESO-1–specific CD4^+^ T cells, a percentage comparable to our prior trial (78%). However, in contrast with our prior study, we did not observe NY-ESO-1–specific CD8^+^ T cells.

**Table 3.** **Effect of DVN therapy on production of NY-ESO-1 specific humoral and cellular immune responses**

Patient	IPSS-R	Best response	Antibody response titer		CD4 response		CD8 response		Post time point (ELISPOT)
			Base	Post	Base	Post	Base	Post	
3DVN	Int	CR	-	-	– (0)	+++ (8)	– (0)	– (0)	Cycle 9, day 1
4DVN	Int	CR	-	-	– (0)	+++ (2)	– (0)	– (0)	Cycle 1, week 8
5DVN	High	SD	-	-	– (0)	+ (2)	– (0)	– (0)	Cycle 9, day 1
6DVN	Very high	SD	-	-	– (0)	– (0)	– (0)	– (0)	Cycle 5, day 1
7DVN	Int	SD	-	-	– (0)	+++ (3)	– (0)	– (0)	Cycle 2, week 6
8DVN	Very high	SD	-	20 075	– (0)	NA	– (0)	– (0)	Cycle 4, day 15

Patient number, IPSS-R, and best response are shown as in Table 1. Immune analyses were performed using peripheral blood samples collected at baseline (Base) and after the EOS (Post). NY-ESO-1 antibody levels were measured using enzyme-linked immunosorbent assay in patient sera. For patients 3DVN and 5-8DVN, assessment of posttreatment antibody titer was performed at cycle 5, day 1. For patient 4DVN, assessment of antibody titer was performed at cycle 1, week 8. Antibody response is displayed as reciprocal titer (negative [−] if reciprocal titer is <100). Frequencies of NY-ESO-1 antigen–specific T cells were measured using ELISPOT assays. Time points of post-EOS samples used in ELISPOT assays are shown. Responses were termed positive when the number of interferon gamma spots per 50 000 cells was twice that of the background level (unpulsed target cells; 21 spots): <25 spots (−); 25 to 99 spots (+); 100 to 199 spots (++); 200 to 499 spots (+++). Numbers in parentheses indicate the number of epitopes recognized by T cells.

### DVN therapy is associated with enrichment of PD-1 blockade gene signature in CD4^+^ T cells

**Figure 2.**
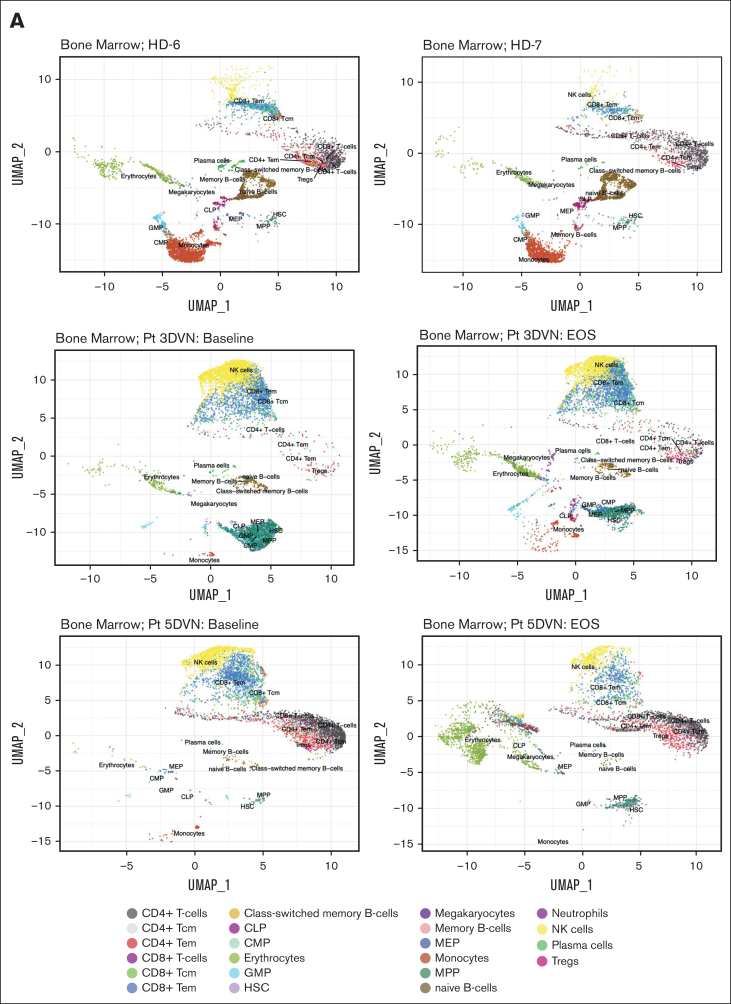

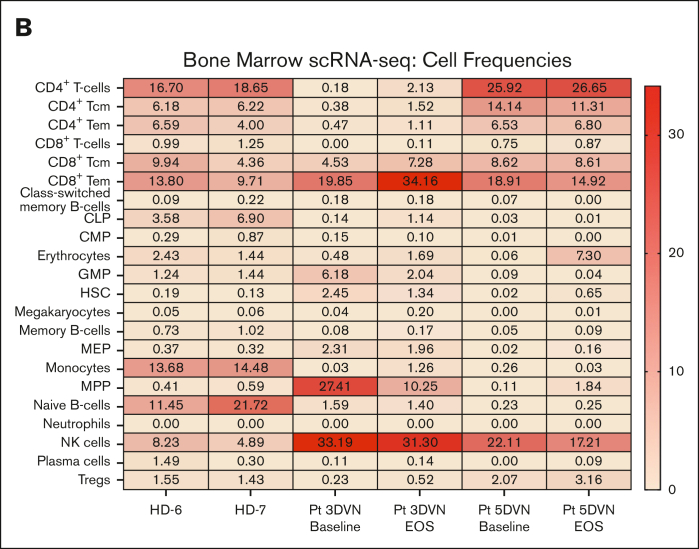
**Effect of DVN therapy on the immunologic milieu*.*** scRNA-seq was performed on bone marrow specimens collected from age-matched HDs and baseline/EOS specimens collected from Pts receiving DVN therapy, DV therapy, or decitabine monotherapy (n = 2 per group). Cells were annotated using SingleR based on a Blueprint-Encode reference database. scRNA-seq data were visualized using UMAP. (A) UMAP visualization plots depicting scRNA-seq data obtained from 2 HDs (top, HD-6 and HD-7) and from baseline/EOS samples from Pts receiving DVN therapy (Pts 3 [middle] and 5 [bottom]). (B) The percentages of annotated cell populations in the bone marrow of HDs and Pts with MDS receiving DVN therapy. CLP, common lymphoid progenitor; CMP, common myeloid progenitor; GMP, granulocyte-monocyte progenitor; HSC, hematopoietic stem cell; MEP, megakaryocyte-erythroid progenitor; MPP, multipotent progenitor; Pt, patient; Tcm, central memory T-cell; Tem, effector memory T-cell; Tregs, -regulatory T-cell; UMAP, uniform manifold approximation and projection.

To characterize the immune response within the MDS microenvironment, we performed scRNA-seq on bone marrow specimens obtained at baseline and at EOS from patients 3DVN and 5DVN. Two bone marrow specimens from age-matched HDs (HD-6 and HD-7) served as a control group. To identify effects that were specific to DVN therapy, we performed scRNA-seq on baseline and EOS bone marrow samples from 2 patients on our previous trial who received only DV (patients 7DV and 9DV) and baseline and post–cycle 4 samples from 2 patients who received HMA monotherapy (1D and 2D). UMAP plots visualizing the results of these analysis are shown in Figure 2A and supplemental Figures 3 and 4.

Overall, we observed similar frequencies of cell populations in HD samples, whereas there was a significant variation among the patients with MDS. (Figure 2B; supplemental Figure 5). We then performed pathway analysis on the CD4^+^ and CD8^+^ T-cell populations. First, we compared CD4^+^ and CD8^+^ T-cell populations in HD samples compared to all MDS baseline samples (supplemental Table 3). Patients with MDS were enriched for pathways and gene signatures associated with inflammation such as interferon gamma responses compared to these cells in HD samples. This may indicate the presence of a proinflammatory environment that is frequently observed in patients with MDS.

**Figure 3.**
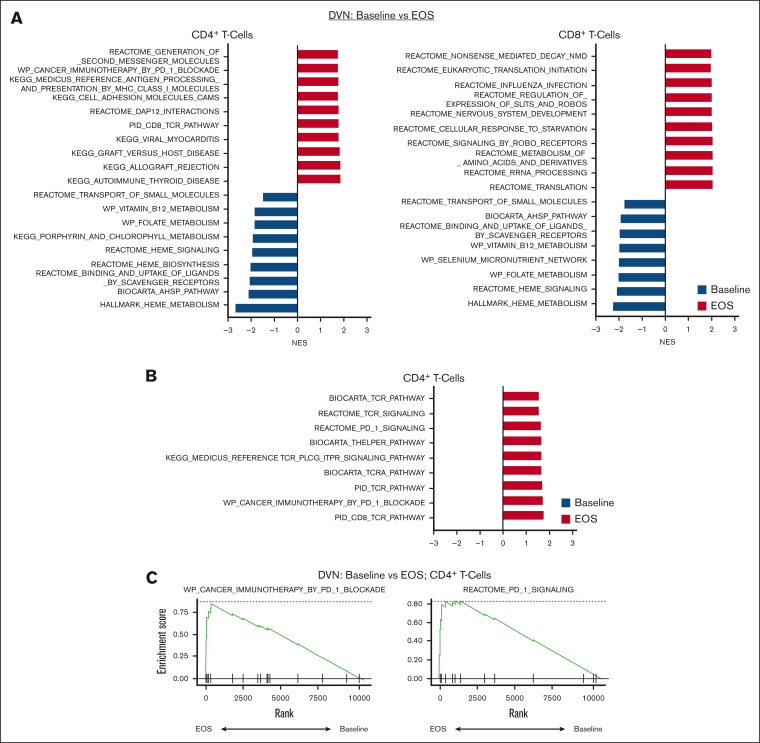
**Enrichment of PD-1 blockade gene signature in CD4^+^ T cells in Pts receiving DVN therapy.** GSEA analysis was performed on pooled CD4^+^ and CD8^+^ T-cell populations as defined by annotation of scRNA-seq data. Significantly enriched pathways were defined based on an adjusted *P* value <.05. For a complete list of significantly enriched pathways, see supplemental Tables 4-7. (A) Top 10 upregulated and downregulated GSEA pathways in baseline vs EOS CD4^+^ T-cell (left) and CD8^+^ T-cell (right) populations in Pts receiving DVN (n = 2). Top 10 pathways were determined based on NESs. Pathways upregulated in samples at baseline (blue) vs EOS (red) are shown with negative or positive NESs, respectively. (B) Upregulated GSEA pathways associated with PD-1 or TCR activity in CD4^+^ T cell at EOS compared with baseline. (C) GSEA plot showing upregulation of the WP_Cancer Immunotherapy by PD-1 Blockade and REACTOME_PD_1_SIGNALING pathways in CD4^+^ T cells at EOS compared with baseline. NES, normalized enrichment score.

We then examined pathways and signatures which were upregulated and downregulated in CD4^+^ and CD8^+^ T-cell populations in patients with MDS at baseline compared to EOS (Figure 3A).

Here, we compared pooled paired samples from patients receiving DVN therapy, DV therapy, or decitabine monotherapy. We acknowledge that any interpretations are limited by sample size and intrapatient variation. In patients receiving DVN therapy, we observed that at the EOS, CD4^+^ cells showed upregulation of multiple pathways associated with PD-1 blockade and T-cell receptor signaling (Figure 3B-C; supplemental Table 4). These pathways were not upregulated in CD8^+^ T cells (Figure 3A; supplemental Table 4). In patients receiving DV or decitabine monotherapy, no such effects were observed in CD4^+^ T cells. (supplemental Tables 5 and 6). Patients receiving decitabine monotherapy showed upregulation of some pathways associated with T-cell activation in CD8^+^ T cells (supplemental Table 6). We also tested whether the combination of decitabine and immunotherapy potentially altered cell-cell interactions between immune effector cells and MDS progenitors using ligand-receptor analysis (LIANA) to identify putative ligand-receptor interactions.^40^ To identify potential interactions which were specific to DVN therapy, we compared patients on this study vs patients receiving decitabine monotherapy. We did not observe any significant interactions that were both post-therapy and combination/DVN specific between T cells and MDS progenitors. LIANA analysis did show a significant interaction between natural killer (NK) cells and MDS progenitors based on expression of *GZMB:CHRM3* and *SPON2:ITGA5* ligand-receptor pairs (supplemental Table 7). Overall, these data suggest that CD4^+^ and CD8^+^ T-cell populations express different signatures associated with T-cell activation in patients with MDS receiving HMAs and immunotherapy.

### Patients with MDS exhibit reduced numbers and expression of costimulatory genes in cDCs

In a previous work, we demonstrated that patients with MDS exhibit decreased numbers of cDC populations characterized by high expression of CD141 (cDC1).^30^^,^^41^

**Figure 4.**
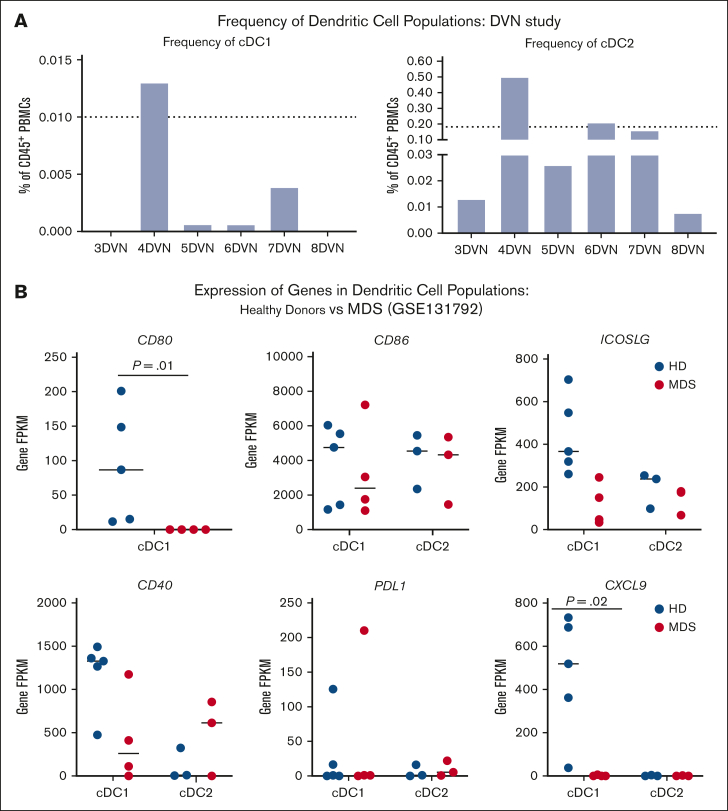
**Pts with MDS exhibit reduced quantity and quality of cDC populations.** (A) Percentage of cDC1 (left) and cDC2 (right) population in the peripheral blood of Pts receiving DVN. Dotted lines depict frequencies of peripheral cDC1 and cDC2 populations in HDs as previously reported.^32^ (B) Expression of cDC genes associated with costimulation (CD80, CD86, ICOSLG, and CD40), exhaustion (PD-L1), and immune cell recruitment (CXCL9) in bone marrow cDC1 and cDC2 populations in HDs (blue circles) vs Pts with MDS (red circles). Expression was quantified using data mined from publicly available bulk RNA sequencing performed on sorted cDC1 and cDC2 populations derived from low cell numbers, as previously reported by Srivastava et al (GSE131792).^41^ FPKM, fragments per kilobase per million mapped fragments; PD-L1, programmed death ligand-1.

Studies in preclinical models of solid tumors have demonstrated a clear link between cDC1s and the response to immunotherapy.^42^, ^43^, ^44^, ^45^ Therefore, we hypothesized that cDC1 quantity and/or quality may impact the immunologic response to decitabine and immunotherapy. We found that the average baseline frequencies of cDC1s and cDC2s in the peripheral blood of patients with MDS receiving DVN therapy were 0.003% and 0.15%, respectively (Figure 4A). By comparison, we previously found that the average frequencies of cDC1s and cDC2s in the peripheral blood of age-matched HDs were 0.01% and 0.18%, respectively.^30^

To see whether cDC quality was impacted in patients with MDS, we examined gene expression of key molecules and factors in cDC1s and cDC2s from patients with MDS. We were unable to unambiguously identify specific cDC populations in scRNA-seq data sets from patients on the DVN study. Therefore, we made use of a previously published data set in which we performed bulk RNA sequencing on cDC1s and cDC2s sorted from HDs and patients with MDS.^41^ In this analysis, which has not been previously published, we focused on expression of costimulatory and inhibitory molecules that are critical for activating an immune response. We found that significantly less *CD80* was expressed in cDC1s from patients with MDS compared to HD (Figure 4B). Based on prior work demonstrating the importance of the CXCL9 chemokine in cDC1-mediated responses to anti–PD-1 therapy, we also examined *CXCL9* expression and observed a significant reduction in *CXCL9* expression in MDS cDC1s compared with HD.^46^^,^^47^ Overall, our data suggest that patients with MDS exhibit decreased numbers and impaired function of cDC1s that may impact the response of the CD8^+^ T-cell compartment to immunotherapy.

## Discussion

The ability of HMA to induce expression of immunogenic tumor antigens and viral mimicry pathways raises the possibility that HMAs could be used to generate effective anti-MDS immune responses. Because higher-risk patients with MDS receive HMAs as standard of care, they are an ideal group for both clinical translation and mechanistic study of these concepts, but a deeper understanding of the immune milieu in MDS is needed to unpick the relative contributions of cytotoxicity, differentiation, and immune response in these patients.

The clinical activity of ICIs in patients with solid tumors have led several groups, including ours, to test the clinical efficacy of these agents in patients with hematologic malignancies. We hypothesized that treatment of patients with MDS with a combination of decitabine, NY-ESO-1 vaccination, and nivolumab would allow us to assess T-cell response in an antigen-specific manner and provide insight into the function of the immune milieu in patients with MDS. In this study, we found that CD4^+^ and CD8^+^ T cells in patients with MDS expressed signatures associated with immune activation. Expression of immune checkpoint molecules PD-1, CTLA-4, T-cell immunoglobulin and mucin domain-containing protein 3 (TIM-3), and T cell immunoglobulin and ITIM domains (TIGIT) on T cells from patients with MDS or AML suggest that T cells in these patient populations are exhausted.^19^^,^^20^^,^^48^^,^^49^ Together, these results suggest a model in which chronic inflammation in MDS results in dysregulation of T-cell responses via upregulation of immune checkpoint molecules.

In our prior clinical trial of NY-ESO-1 vaccination plus decitabine, we observed NY-ESO-1–specific immune responses in both CD4^+^ and CD8^+^ T cells. This contrasts with the current study in which we observed NY-ESO-1–specific immune responses in CD4^+^ but not CD8^+^ T cells. One potential explanation for this is the variable induction of NY-ESO-1 expression. Seven of 9 patients enrolled in our prior clinical trial of NY-ESO-1 vaccination plus decitabine exhibited induction of NY-ESO-1 expression during the first cycle of therapy.^30^ In this study, only 1 of 7 showed detectable induction of NY-ESO-1 expression during the first cycle of therapy and 3 of 7 patients exhibited NY-ESO-1 expression at any time during study therapy.

Prior studies have shown that vaccination against NY-ESO-1, as used in this study, can produce antigen-specific CD4^+^ and CD8^+^ T-cell responses and that such responses may be augmented by inhibition by PD-1.^36^^,^^50^^,^^51^ The differences in observed CD4^+^ vs CD8^+^ T-cell responses in this study, compared to those seen in our prior work, underscores the potential for intrapatient heterogeneity to impact immune responses.^30^ In addition to variable induction of NY-ESO-1 expression, this could be due to multifactorial causes including defects in antigen processing and presentation, decreased ability of T cells to respond to antigenic stimulation, and immune suppressive effects of decitabine.^13^^,^^52^ Additional studies of functionally defined T-cell populations (eg, central and effector memory) and the T-cell repertoire in patients with MDS through T-cell receptor sequencing may shed further light on this subject.^53^^,^^54^

We have reported in a large cohort (n = 71) that patients with MDS exhibit fewer numbers of cDC1s and that the decreased population is associated with reduced survival.^41^ In our prior clinical study, frequency of cDC1s (>0.001% in 3/8 patients) in the peripheral blood was associated with greater vaccine response.^30^ In this study, peripheral cDC1 frequencies were comparable to those observed in our previous study (>0.001% in 4/6 patients) with 1 patient (4DVN) exhibiting a cDC1 frequency comparable to a heathy individual (Figure 4A). Based on these results, and those of multiple preclinical and clinical studies in solid tumors that cDC1 frequency and function govern responses to anti–PD-1 and other immunotherapies, we hypothesize that characteristics of this population in patients with MDS could contribute to clinical responses to immunotherapy.^31^^,^^42^, ^43^, ^44^, ^45^

In a new analysis of gene expression data previously published by our group, we found that cDC1s in patients with MDS show significantly decreased expression of *CD80* and *CXCL9*. Decreased expression of CXCL9 may be especially important as expression of this chemokine by cDC1s in the tumor microenvironment is necessary for successful anti–PD-1 therapy.^46^^,^^47^ A decrease in cDC1 number or function associated with MDS could explain why patients 3DVN and 4DVN, who had undetectable and high numbers cDC1s respectively, both showed no induction of CD8^+^ T cells. Although our study focused on the cDC1 population, a recent study has shown that cDC2 function is also impaired in patients with MDS.^55^ In a retrospective analysis, some patients with melanoma who responded to anti–PD-1 therapy exhibited high numbers of cDC2s and low numbers of cDC1s, suggesting the possibility that the cDC2 compartment may also contribute to clinical response to ICIs.^56^ In patients enrolled in this study, cDC2 frequencies were comparable to those observed in HDs (Figure 4A). Further work is warranted to elucidate the function of cDC1 and cDC2 populations in the MDS microenvironment and how to use this knowledge to identify an optimal approach to activate an antitumor immune response.

Preferential activation of CD4^+^ T-cell responses is supported by our observation that a gene signature associated with blockade of the PD-1 pathway was upregulated only in CD4^+^ memory T cells but not in CD8^+^ T cells. We acknowledge that these observations and our interpretations are limited to the small number of patients enrolled on study and that additional studies are needed to confirm that activation of T cells are preferential to CD4^+^ T cells compared to the CD8^+^ T cells. However, these data are similar to those reported in other early phase trials combining HMAs with ICIs which have resulted in markedly variable clinical and immunologic responses.^21^^,^^57^^,^^58^ Our results also suggest the possibility that immunotherapy in patients with MDS may influence interactions between NK cells and MDS progenitors which may indicate an additional avenue for further study of combining HMAs with agents that target NK cells such as trispecific killer engagers.^59^

In conclusion, this trial has revealed the challenges of activating tumor antigen–specific immune responses in MDS. We propose that assessment of immune responses in a tumor antigen–specific manner is required to optimize immunotherapies for these patients. Such assessments may include measurement of antigen expression, quantification of APC and T-cell populations, and functional characterization of APCs during therapy to optimize the delivery of cancer vaccines. In addition, a deeper understanding of the impact of HMA therapy, including potential differences in using decitabine vs azacitidine, on the interaction between immune cells and MDS progenitors is necessary to combine chemotherapy with immunotherapy in this patient population. These studies will significantly impact the clinical management of MDS and other myeloid cancers through development of novel combination approaches for modulating the immune system to enhance the efficacy of epigenetic and immunotherapy in MDS and myeloid cancers.

Conflict-of-interest disclosure: E.A.G. reports receiving honoraria for CME from AAMDS, MedscapeLIVE!, MediCom Worldwide, Inc, MJH Life Sciences, The American Society of Hematology, MDS International Foundation, and Physicians Educational Resource; providing consultancy for AbbVie, Alexion Pharmaceuticals, Apellis Pharmaceuticals, Takeda Oncology, Astex Pharmaceuticals/Taiho Oncology, Alexion Pharmaceuticals/AstraZeneca Rare disease, Celgene/Bristol Myers Squibb, CTI BioPharma, Novartis, Partner Therapeutics, Picnic Health, and Servier; and receiving research support from Alexion Pharmaceuticals, Apellis, Astex/Otsuka Pharmaceuticals, Blueprint Medicines, Celldex Therapeutics, Genentech Inc, and NextCure, Inc. K.O. is a cofounder of Tactiva Therapeutics. M.J.N. reports receiving research support from Imago Biosciences. The remaining authors declare no competing financial interests.

## Supplementary Material

Supplemental Methods, References, Figures, and Tables Legends
