# Supplementary material for: Checkpoint immunotherapy is associated with preferential activation of tumor antigen–specific CD4+ T cells in MDS
Source: Blood Neoplasia. 2025 Apr 25;2(3):100106. doi: 10.1016/j.bneo.2025.100106 (PMC12343363; doi:10.1016/j.bneo.2025.100106)
Supplement: Supplemental Methods, References, Figures, and Tables Legends [file BNEO_NEO-2024-000457-mmc1.pdf]

Supplemental Methods

Analysis of NY-ESO-1 Promoter Methylation and Expression

Methylation of the *NY-ESO-1* promoter in CD11b$^+$ cells was determined by quantitative sodium bisulfite pyrosequencing as previously described.[1,2]. Genomic DNA was isolated and sodium bisulfite conversion performed using the EZ DNA Methylation Kit (Zymo Research). PCR reactions were performed using biotinylated primers on sodium bisulfite–converted DNA under the following conditions: 95°C for 30 s, 55.7°C for 30 s, and 72°C for 1 min, for 45 cycles. Biotinylated PCR products were bound to Streptavidin Sepharose High Performance beads (Amersham Biosciences) and purified using the Pyrosequencing Vacuum Prep Tool (Biotage AB). Pyrosequencing of purified single-stranded PCR products was performed using the PSQ HS96 Pyrosequencing System (Biotage AB). For each time-point, pyrosequencing was performed on technical duplicates. Human OVCAR cells and HCT116 cells deficient in DNMT1/3b served as a positive and negative controls for NY-ESO-1 methylation receptively. To measure NY-ESO-1 expression, RNA and cDNA was prepared from CD11b$^+$ cells isolated from PBMCs and reverse transcriptase nested PCR (RT-PCR) was performed using two reactions as previously described.[3] The first reaction amplified a 332 bp followed by a second reaction that amplified a 177 bp product. PCR products were amplified with initial denaturation at 95˚C for 5 min, followed by 35 cycles of denaturation at 95˚C for 30 sec., annealing at 60˚C for 30 sec., and extension at 72˚C for 30 sec., followed by a final 5 min extension at 72˚C. PCR products were analyzed on 2% agarose gel by ethidium bromide staining. All primer sequences are shown in **Supplementary Table 8.**

Analysis of NY-ESO-1 Specific Immune Responses.

ELISA Assay

NY-ESO-1 protein (provided by the Ludwig Institute for Cancer Research) was adsorbed at 0.5 µg/ml DPBS to the wells of half-area ELISA plates (Corning). Plates were incubated overnight at 4˚ and blocked for 2 hrs at room temperature (RT) in 0.05% Tween-20/5% non-fat (NF) milk in DPBS. Serially dilutions of sera (diluted in 0.05% Tween-20/5% NF milk) were added to the plates and incubated overnight at 4˚. Samples were tested over a range of 1:400 to 1:409,600. After washing, a secondary antibody solution (goat anti-human IgG-AP diluted at 1:10,000 in 0.05% Tween-20/5% NF milk, Southern Biotechnology) was added and incubated for 1 hr at RT. Plates were washed and incubated with AttoPhos substrate solution (Promega) for 30 minutes at RT

in the dark. Reactions were stopped using 3N NaOH and plates were immediately read using a BioTek Synergy HT microplate reader.

ELISPOT Assay

$CD4^+$ and $CD8^+$ T-lymphocytes were isolated using antibody-coated magnetic beads and cultured in RPMI medium 1640 supplemented with 10% human AB serum (NABI, Boca Raton, FL), L-glutamine (2 mM), penicillin (100 units/ml), streptomycin (100 µg/ml). To generate APCs for T-cell pre-sensitization, autologous T-cell depleted PBMCs were pulsed overnight in serum-free media (X-VIVO-15; BioWhittaker) with a pool of synthetic overlapping 20- to 25-mer peptides that cover the entire NY-ESO-1-protein. Pulsed APCs were irradiated and co-cultured with T cells at a 2:1 ratio. Cultures were supplemented with IL-2 (10 units/ml; Roche Molecular Biochemicals) and IL-7 (20 ng/ml; R & D Systems) and fresh cytokines were added every 3 days. After 14 days in culture, pre-sensitized T-cells were harvested for ELISPOT analysis. T cells were stimulated overnight with T-APCs (autologous $CD4^+$ T cells activated with PHA) pulsed with individual NY-ESO-1 peptides on ELISPOT plates pre-coated with anti-human IFN-γ mAb (Clone 1-D1K, Mabtech). Cells were washed off and the plate was incubated for 2 hrs with anti-human IFN-γ mAb (Clone 7-B6-1, Mabtech, 1:5,000 dilution) followed by another washing and incubation for 1 hr with Streptavidin-ALP (Mabtech, 1:1,000 dilution). BCIP/NBT substrate (Sigma) was used to visualize IFNg spots. Responses were scored positive when spot numbers in the presence of NY-ESO-1 peptide-pulsed target cells were >20 spots/50,000 cells) and were at least 2 times more than that spot count of peptide un-pulsed target cells.

Gene Mutation Analysis. Cryopreserved PBMCs from selected time-points were thawed and high-quality genomic DNA (gDNA) was extracted using the All-Prep kit (Qiagen) following manufacturer recommendations. DNA concentration was determined using the Qubit HS dsDNA assay on a Qubit 2.0 Flourometer (Thermo Fisher Scientific). Error-corrected targeted next-generation sequencing was performed on gDNA from PBMCs collected before and after treatment using a custom anchored multiplex PCR based targeted DNA sequencing panel (VariantPlex, ArcherDx, Boulder, CO) designed with coverage of 29 commonly mutated genes in AML/MDS as previously described.[4] In short, 200ng of gDNA was subjected to library generation and paired-end 150bp sequencing on a Novaseq 6000 (Illumina). Raw sequencing fastq files were analyzed using the

Archer Analysis software version 6.0.2.3 and residual variants identified down to a variant allele fraction of 0.1% or greater. Raw sequencing data are available in the NCBI Sequence Read Archive (SRA) (Accession: PRJNA1111797).

Single Cell RNA Sequencing

Single cell libraries were generated using the 10x Genomics platform using Single Cell 3' Kit v3. Bone marrow cell suspensions from healthy age-matched donors and patients were assessed with ViaStain AOPI using a Cellometer K2 automated cell counter (Nexcelom), to determine concentration, viability and the absence of clumps and debris that could interfere with single cell capture. Cells were loaded into the Chromium Controller (10X Genomics) where they were partitioned into nanoliter-scale Gel Beads-in-emulsion with a single barcode per cell. Reverse transcription was performed, and the resulting cDNA was amplified. The full-length amplified cDNA was used to generate gene expression libraries by enzymatic fragmentation, end-repair, a-tailing, adapter ligation, and PCR to add Illumina compatible sequencing adapters. The resulting libraries were evaluated on D1000 screentape using a TapeStation 4200 (Agilent Technologies) and quantitated using Kapa Biosystems qPCR quantitation kit for Illumina. Libraries were pooled, denatured, and diluted to 300pM with 1% PhiX control library added. The resulting pool was then loaded into the appropriate NovaSeq Reagent cartridge and sequenced on a NovaSeq6000 following the manufacturer's recommended protocol (Illumina Inc.).

Bioinformatic Analysis of sc-RNA Sequencing Data

Raw sequencing data were processed using Cell Ranger (CR) (v6.0.0) pipeline to generate fastq files and count matrices. Fastq files were preprocessed and then reads were aligned and quantified generating feature-barcode count matrices. Gene-barcode matrices containing barcodes with the Unique Molecular Identifier (UMI) counts were filtered using CR's cell detection algorithm. Downstream analyses were performed mainly using Seurat (v4.1.0) single-cell analysis R package.[5] 14 single-cell RNA seq samples (~6900 cells per sample) were individually read into a Seurat object to examine feature number, mitochondrial percentage and read count distributions within sample. Cells with less than 300 features or >10% mitochondrial content were filtered out. DoubletDecon (v1.1.4) was used to detect and remove doublets.[6] SingleR (v1.6.1) was used to

annotate individual immune cell types using Blueprint-Encode database.[7] Individual samples are then merged into a single Seurat object. Data normalization and scaling were performed using Seurat's SCTransform function regressing against mitochondrial percentage. Data were then dimension-reduced via UMAP and clustered (Louvain algorithm) for downstream visualization. Differential gene expression analysis was performed using Seurat's FindMarkers function selecting test.use="negbinom", fitting a two-group negative-binomial generalized linear model (GLM). Statistical tests were performed selecting targeted immune cell populations and then comparing between patient groups and treatments accounting for subject as fixed effect. Subsequent gene-set enrichment analysis is performed on comparisons of interest using GSEApreranked procedure from GSEA (v3.0beta).[8] Negative log-pvalues are multiplied by the sign of the log2FC are entered as ranked list input. Hallmark, C2CP, C6 and C7 gene symbol sets from MSigDB (v7.1) were employed for these analyses. Significant pathways are defined as those with FDR < 0.1 and the complete list of pathways are shown in **Supplemental Tables 3-6..**

Bioinformatic Cell Communication Analysis

To perform a cell communication analysis, we employed LIANA v0.1.3 (LIgand-receptor ANalysis frAmework), a consensus tool for predicting ligand-receptor interactions from scRNA-seq data[9], to predict signaling interactions between HSPC (HSC, CLP, MEP, CMP, GMP, MPP) and immune (NK cells, CD4+ T-cells, CD8+ T-cells) populations in patients treated with combination therapy (n=2) or monotherapy (n=2). All analysis was performed using default parameters. Only interactions with an “aggregate_rank” rank statistic less than 0.05 were considered significant and used in downstream analyses. The ligand-receptor interactions were further filtered to only include those that were post-therapy-specific for a given patient (*i.e.* existing in the post-therapy sample for a patient but not the pre-therapy sample). Interactions which were specific to the combination therapy or monotherapy group are highlighted and a list of LIANA-predicted interactions is available in **Supplementary Table 7**.

Dendritic Cell Phenotyping.

Dendritic Cell (DC) immunophenotyping of patients enrolled on study was performed by staining PBMCs or BM for 30 minutes on ice with a cocktail of primary antibodies and secondary reagents listed in **Supplemental**

Table 9.[10] Gating strategies for DC immunophenotyping are shown in **Supplemental Figure 6**. Immunophenotyping was performed using an LSRII (Becton Dickinson) and all raw flow cytometry data were analyzed using FlowJo v.10.2 software (TreeStar). Data obtained from bulk RNA-sequencing of sorted bone marrow conventional DC populations were previously reported and are available at GEO under accession number GSE131792.[11]

Supplemental Methods References

1. Srivastava P, Paluch BE, Matsuzaki J, et al. Immunomodulatory action of the DNA methyltransferase inhibitor SGI-110 in epithelial ovarian cancer cells and xenografts. *Epigenetics*. 2015;10(3):237–246.
2. Woloszynska-Read A, Mhawech-Fauceglia P, Yu J, Odunsi K, Karpf AR. Intertumor and intratumor NY-ESO-1 expression heterogeneity is associated with promoter-specific and global DNA methylation status in ovarian cancer. *Clin Cancer Res*. 2008;14(11):3283–3290.
3. Srivastava P, Paluch BE, Matsuzaki J, et al. Induction of cancer testis antigen expression in circulating acute myeloid leukemia blasts following hypomethylating agent monotherapy. *Oncotarget*. 2016;7(11):12840–12856.
4. Dillon LW, Gui G, Logan BR, et al. Impact of Conditioning Intensity and Genomics on Relapse After Allogeneic Transplantation for Patients With Myelodysplastic Syndrome. JCO Precis Oncol. 2021;5:PO.20.00355.
5. Satija R, Farrell JA, Gennert D, Schier AF, Regev A. Spatial reconstruction of single-cell gene expression data. *Nat Biotech*. 2015;33(5):495–502.
6. DePasquale EAK, Schnell DJ, Van Camp P-J, et al. DoubletDecon: Deconvoluting Doublets from Single-Cell RNA-Sequencing Data. *Cell Reports*. 2019;29(6):1718–1727.e8.
7. Aran D, Looney AP, Liu L, et al. Reference-based analysis of lung single-cell sequencing reveals a transitional profibrotic macrophage. *Nature Immunology*. 2019;20(2):163–172.
8. Subramanian A, Tamayo P, Mootha VK, et al. Gene set enrichment analysis: A knowledge-based approach for interpreting genome-wide expression profiles. *Proc Natl Acad Sci U S A*. 2005;102(43):15545–15550.
9. Dimitrov D, Türei D, Garrido-Rodriguez M, et al. Comparison of methods and resources for cell-cell communication inference from single-cell RNA-Seq data. *Nat Comm*. 2022;13(1):3224–13.
10. Griffiths EA, Srivastava P, Matsuzaki J, et al. NY-ESO-1 Vaccination in Combination with Decitabine Induces Antigen-Specific T-lymphocyte Responses in Patients with Myelodysplastic Syndrome. *Clin Cancer Res*. 2018;24(5):1019–1029.
11. Srivastava P, Tzetzo SL, Gomez EC, et al. Inhibition of LSD1 in MDS progenitors restores differentiation of CD141(Hi) conventional dendritic cells. Leukemia. 2020;34(9):2460-2472.

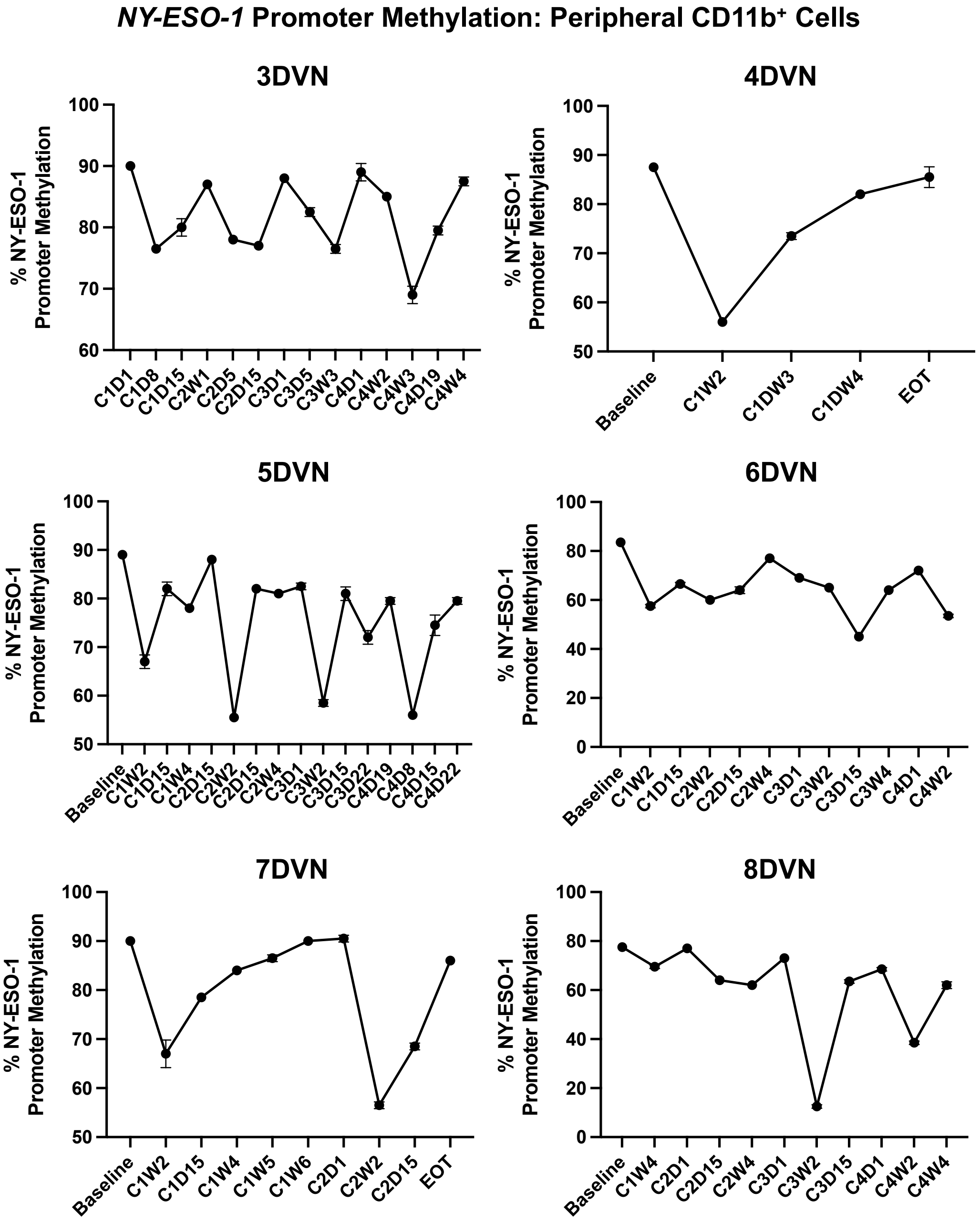

Supplemental Figure 1. Effect of DVN therapy on methylation status of *NY-ESO-1*. Pyrosequencing was performed on DNA extracted from peripheral CD11b$^+$ blood cells serially collected from patients receiving decitabine, vaccine, and nivolumab (DVN) therapy to determine percentage of methylated DNA. For each patient, the percentage of methylated *NY-ESO-1* promoters in CD11b$^+$ cells (average of 2 technical replicates) at pre-treatment and at serial time points during treatment is shown. C = decitabine cycle number; D = day of each cycle. Each individual cycle has a range of 1 to 28 with decitabine treatment occurring on days 1 – 5.

NY-ESO-1 Gene Expression: Peripheral CD11b$^+$ Cells

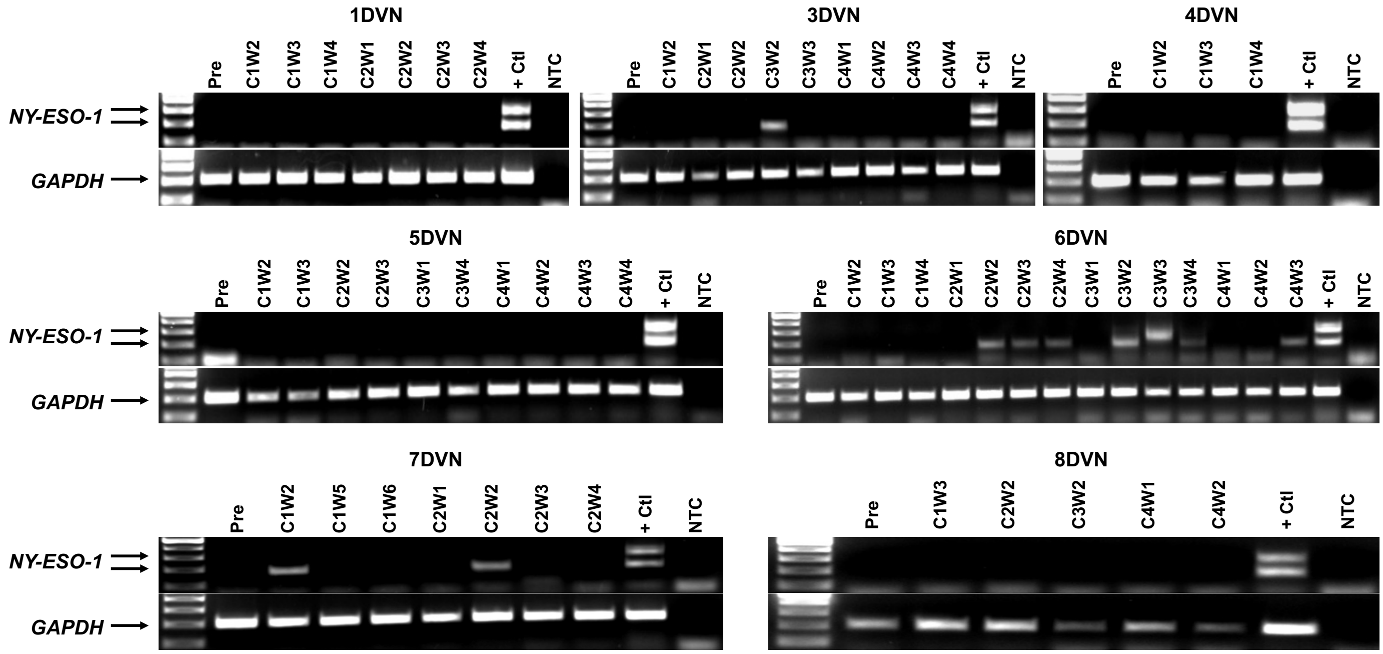

Supplemental Figure 2. Effect of DVN therapy on *NY-ESO-1* expression. RNA was extracted from peripheral CD11b$^+$ cells serially collected from patients receiving decitabine, vaccine, and nivolumab (DVN) therapy. Nested RT-PCR was performed to evaluate *NY-ESO-1* expression. *GAPDH* was used as the loading control. C = decitabine cycle number; D = day of each cycle. Decitabine-treated OVCAR cells served as the positive control (+ Ctl); no template was used as the negative control (NTC).

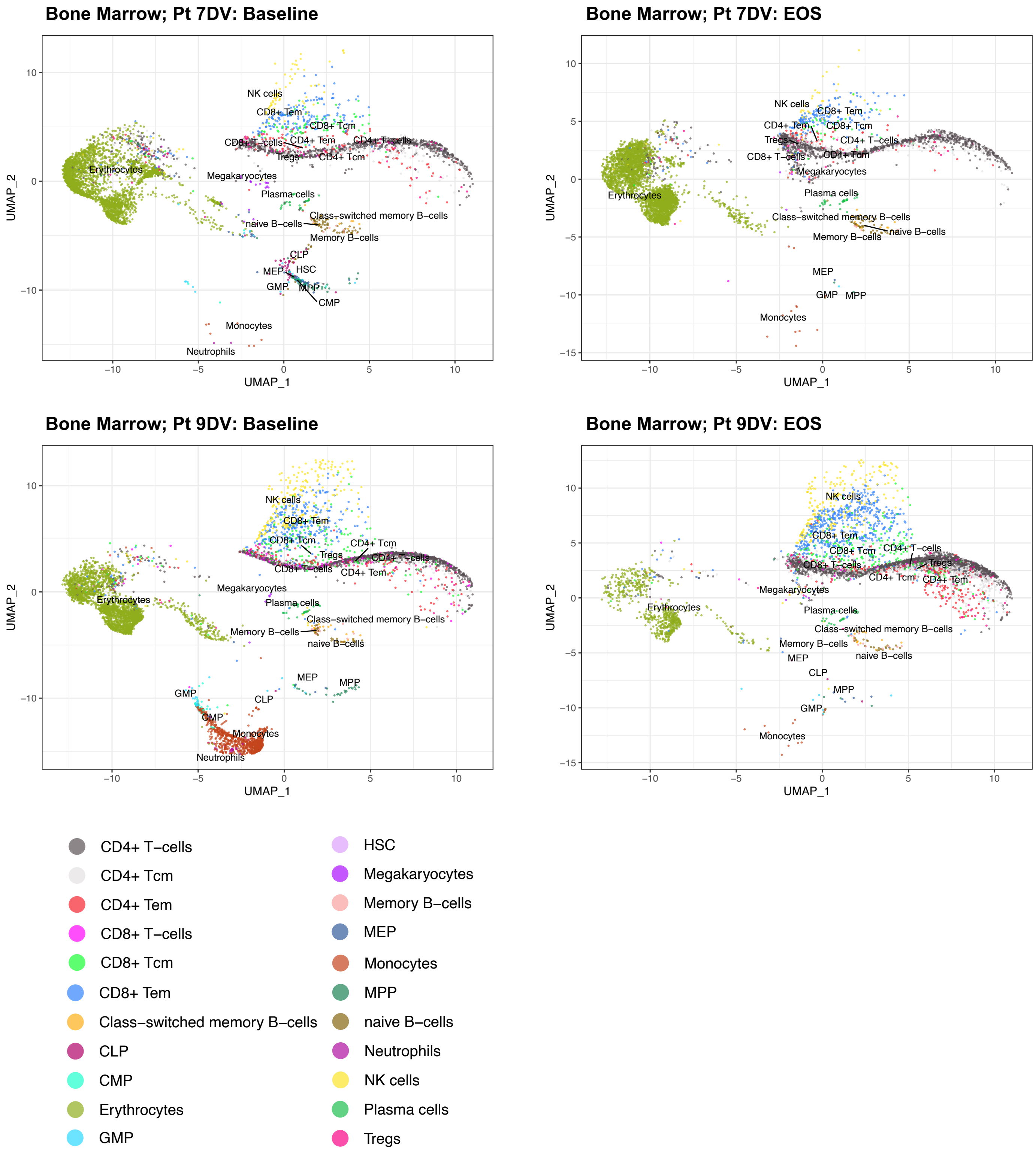

Supplemental Figure 3. Related to Effect of DVN therapy on the immunologic *milieu.* UMAP visualization plots depicting single-cell RNA (scRNA) sequencing data obtained from bone marrow specimens collected at baseline and end-of-study (EOS) from patients receiving decitabine and NY-ESO-1 vaccination (7DV and 9DV). Patient ID numbers correspond to those previously reported.[10]

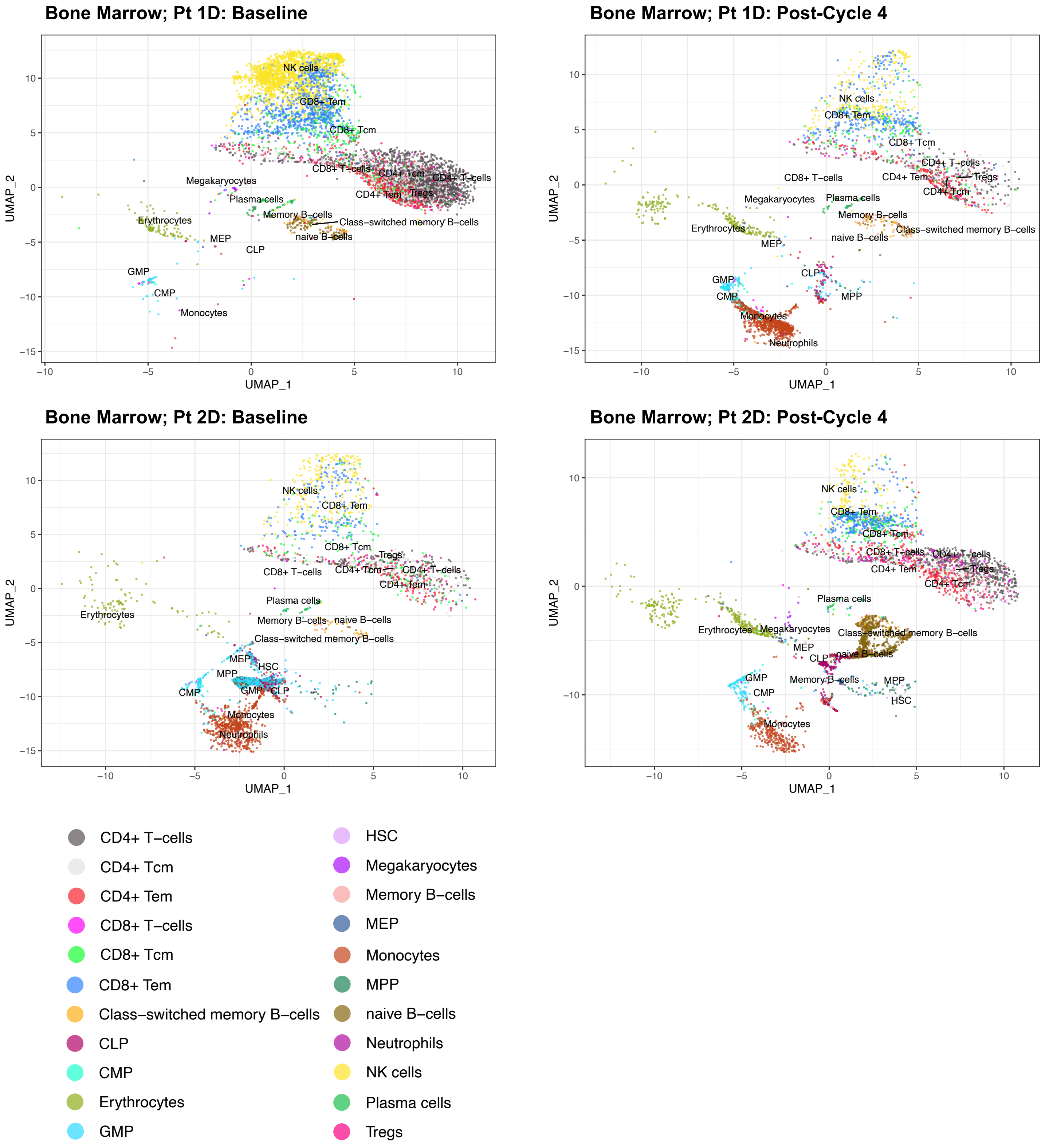

Supplemental Figure 4. Related to Effect of DVN therapy on the immunologic *milieu*. UMAP visualization plots depicting single-cell RNA (scRNA) sequencing data obtained from bone marrow specimens collected at baseline and post-cycle 4 (PC4) from patients receiving decitabine monotherapy (1D and 2D) (n = 2). Cells were annotated using SingleR based on a Blueprint-Encode reference database.

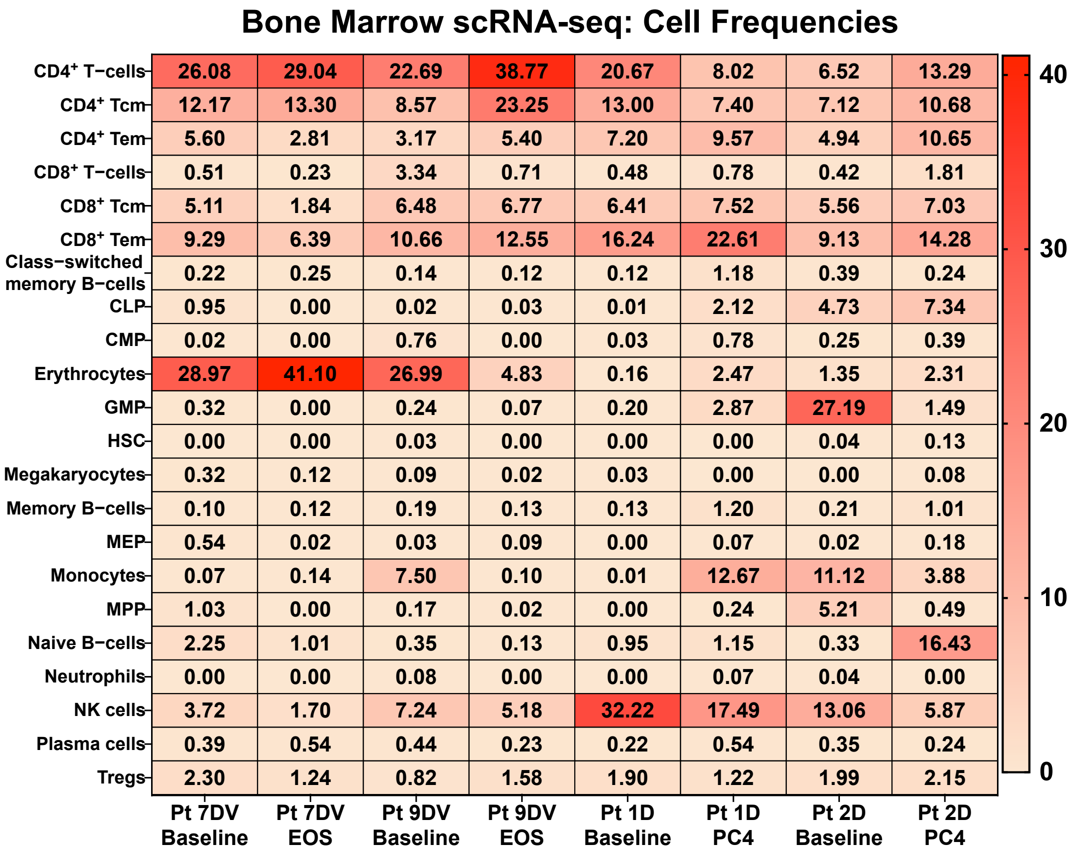

Supplemental Figure 5. Related to Effect of DVN therapy on the immunologic *milieu.*

The percentages of annotated cell populations in the bone marrow of MDS patients receiving decitabine *plus* vaccination (7DV and 9DV) or decitabine monotherapy (1D and 2D) at baseline and end-of-study (EOS; for 7DV and 9DV) or after post-cycle 4 (PC4; for 1D and 2D) Cell phenotypes were determined based on annotation of single-cell RNA sequencing data using the Blueprint-Encode reference database.

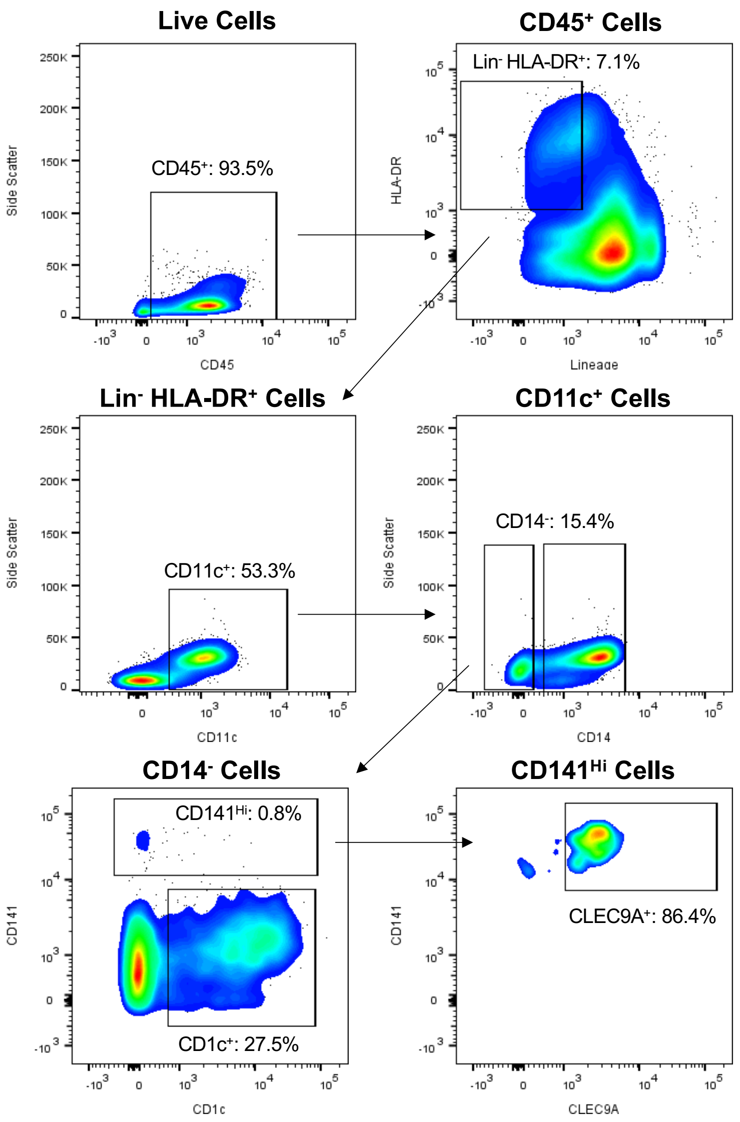

Supplemental Figure 6. Gating strategy to identify conventional DC subsets. Representative plots showing gating strategy to identify conventional DC (cDC) populations for all samples. Live single cells were first gated based on CD45 (top left). $CD45^+$ cells were then gated based on a Lineage-negative (Lin^-), HLA-DR^+ immunophenotype (top right). The cDC population was defined as positive for CD11c expression ($CD11c^+$, middle left) and then negative for CD14 expression ($CD14^-$, middle right). The cDC subsets were gated as $CD141^{Hi}$ and $CD1c^+$ (bottom left). Due to the relative rarity of $CD141^{Hi}$ cDCs in MDS/AML samples, CLEC9A staining was used to confirm the presence of the $CD141^{Hi}$ cDC population (bottom right). Numbers indicate the percentage of gated cells within the parent population.

Supplemental Table Legends

Supplemental Table 1. Complete description of cytogenetics and long-term clinical follow-up of patients enrolled on NCT0335871.

Supplemental Table 2. Mutational profiling of patients receiving DVN therapy. Patient samples are marked by ID number. VAF = variant allele fraction; BM = bone marrow; AA = amino acid; EOS = end of study.

Supplemental Tables 3-6. GSEA Pathways significantly enriched in healthy donor and MDS patient T cell populations. These tables list GSEA pathways enriched in $CD8^+$ and $CD4^+$ T-cell populations based on the following comparisons:

Supplemental Table 3: Healthy donors *versus* MDS patients at baseline.

Supplemental Table 4: MDS patients receiving decitabine, vaccination, nivolumab (DVN): baseline *versus* end of study.

Supplemental Table 5: MDS patients receiving decitabine plus vaccination (DV): baseline *versus* end of study.

Supplemental Table 6: MDS patients receiving decitabine monotherapy: baseline *versus* post-cycle 4.

All pathways shown in tables were identified using Gene Set Enrichment Analysis with an adjusted p-value < 0.05.

Supplemental Table 7. Predicted interactions between hematopoietic stem and progenitor cells (HSPC) and immune effector populations. LIANA analysis was performed to predict significant interactions between HSPCs and $CD8^+$/$CD4^+$ T cell or natural killer (NK) cell populations based on expression of ligand-receptor pairs. Analysis was performed on paired samples collected from patients receiving decitabine monotherapy or decitabine, vaccination, nivolumab (DVN). Populations under the “source” column are expressing the ligand and populations under “target” are expressing the receptor. Interactions predicted to be specific to the DVN samples are highlighted and marked as “TRUE” in the “combination.specific” column.

Supplemental Table 8. Primers used in quantitative pyrosequencing and nested RT-PCR experiments.

Supplemental Table 9. Antibodies used in flow cytometry experiments
